# Assessment of cytotoxicity and genotoxicity of stem bark extracts from *Canarium odontophyllum* Miq. (dabai) against HCT 116 human colorectal cancer cell line

**DOI:** 10.1186/s12906-016-1015-2

**Published:** 2016-01-29

**Authors:** Dayang Fredalina Basri, Zafira Ayushah Zainul Alamin, Kok Meng Chan

**Affiliations:** 1 School of Diagnostic and Applied Health Sciences, Faculty of Health Sciences, Universiti Kebangsaan Malaysia, Jalan Raja Muda Abdul Aziz, Kuala Lumpur, 50300 Malaysia; 2 Toxicology Laboratory, Faculty of Health Sciences, Universiti Kebangsaan Malaysia, Jalan Raja Muda Abdul Aziz, Kuala Lumpur, 50300 Malaysia

**Keywords:** *Canarium odontophyllum*, Dabai, Colorectal cancer, HCT 116, CCD-18co, Cytotoxicity, Apoptosis, DNA damage

## Abstract

**Background:**

*Canarium odontophyllum* Miq. is a plant species widely known as ‘dabai’ and can be vastly found in Sarawak. The aim of this study was to assess the cytotoxic and genotoxic effect of extracts from stem bark of *C. odontophyllum* against HCT 116 human colorectal cancer cell line.

**Method:**

The IC_50_ values of the aqueous, methanol, and acetone extracts against HCT 116 cells as well as the acetone extract against human colon fibroblast cell line CCD-18co were determined using the MTT assay. The concentration of the extracts ranged from 12.5 to 200 μg/ml at treatment time of 24, 48 and 72 h. Annexin V-FITC/PI labelling assay was employed to determine mode of HCT 116 cell death induced by acetone extract at 48 h. The DNA damage induced by the extract in HCT 116 cells was detected using alkaline comet assay at 30 min of IC_10_ and IC_25_ treatment.

**Results:**

Acetone extract exhibited the highest cytotoxic effect against HCT 116 cells compared to methanol and aqueous extract at 24, 48 and 72 h. Despite no cytotoxic effect by acetone extract against CCD-18co cells at 24 and 48 h, however at 72 h, CCD-18co cells proliferated. Apoptosis assessment using Annexin V-FITC/PI labelling assay revealed that the primary cell death was via apoptosis after 48 h treatment. Low doses of acetone extract from stem bark of *C. odontophyllum* showed significant DNA damage in HCT 116 cells with tail moment of 6.187 ± 0.718 A.U and 7.877 ± 0.142 A.U, respectively.

**Conclusions:**

Acetone extract from stem bark of *C. odontophyllum* has high potential in the development of anticancer agent against HCT 116 cells with no cytotoxic effect against human colon fibroblast cells.

## Background

Colorectal cancer or colon cancer is defined as an uncontrollable cell growth at the lower part of the digestive system that is the large intestine [1]. Colorectal cancer is one of the main causes to high mortality and morbidity rate of cancer. Moreover, colorectal cancer is more frequent in the industrialized world than in developing countries [2]. Colorectal cancer falls in second highest mortality and morbidity rate followed by lung cancer, gastric cancer and breast cancer. In Malaysia, colorectal cancer mostly occurs in male and third in frequency among females. Colorectal cancer is common among the age group of 50 years old and above [3].

Plant derived anti-cancer agents play an important role in cancer chemotherapy [4]. Additionally, plant derived anti-cancer agents are known to be safer and give less side effects when in comparison with those synthetic anti-cancer agents available [5]. *Canarium odontophyllum* Miq. is a type of plant that is known as ‘dabai’ or ‘Borneo olive’. It can be found in Sarawak, Malaysia especially in Sibu, Sarikei and Kapit [6]. It belongs in the Burseraceae family and *Canarium* L. genus [7]. The fruit of *C. odontophyllum* is oval with a purplish skin and has a single seed along with a hard and thick endocarp [8]. Almost all parts of the plant were tested for medical researches including the fruit, peel, shell of the seed, pulp, leaf and stem bark. The pulp extract from *C. odontophyllum* fruit was found to inhibit the growth of *Candida glabrata* [9]. The leaf and shell extracts from *C. odontophyllum* were shown to have antimicrobial activity against a wide range of pathogenic bacteria [10] whereas both the leaf and stem bark of *C. odontophyllum* demonstrated promising anticancer property [11]. However, previous study merely reported preliminary screening of cytotoxic activity against human colorectal carcinoma HCT 116 cell line attributed to the presence of flavonoid, tannin, saponin, terpenoid and phenolic compound [12]. Damage to DNA always occurs from endogenous and exogenous agents such as reactive oxygen species (ROS) from cellular metabolism and ultraviolet light from the sun [13]. Chemical carcinogens, radiation and genotoxic anti-cancer agents can cause DNA damage [14]. When there is DNA damage, the damage itself will cause cell cycle arrest where it can lead to DNA repair or cell death via apoptosis [15]. Therefore the objective of the present study is to investigate the mechanism of cell death and to determine the genotoxic effect of extracts from the stem bark of *C. odontophyllum* against HCT 116 human colorectal cancer cell line.

## Methods

### Plant material

Stem bark of *Canarium odontophyllum* Miq. was obtained from Sarawak, Malaysia. All plant parts were identified and authenticated by Mr. Sani Miran and deposited in the Herbarium of the Universiti Kebangsaan Malaysia (UKM), Bangi, Selangor, Malaysia with a voucher specimen number of UKMB 40052.

### Preparation of plant extracts

The stem bark of *Canarium odontophyllum* was extracted in three different solvents with different degree of polarity namely acetone, methanol and aqueous. To prepare a stock extract solution of 100 mg/ml, 100 mg of acetone and methanol extract were dissolved with 1 ml of 100 % dimethyl sulfoxide (DMSO) whereas for aqueous extract, 1 ml of distilled water was used as the diluent. The solution was mixed well with an autovortex until the solution was completely dissolved. All extracts were sterilized by passing through a 0.22 μM membrane filter and were stored in air-tight jars at −20 °C refrigerator until further use.

### Preparation of cell culture

HCT 116 and CCD-18co were obtained from American Type Culture Collection (ATCC) (Rockville, MD USA). HCT 116 cell line (ATCC Number: CCL-247™) was cultured in McCoy 5A media (1x) (Sigma Aldrich, USA) whereas the normal human colon cell line, CCD-18co (ATCC Number: CRL-1790™) was cultured in EMEM (Eagle’s Minimum Essential Medium) (1x) (Sigma-Aldrich, USA). Culturing of HCT 116 and CCD-18co were carried out in a sterile laminar flow chamber to avoid any possible contamination. McCoy 5A and CCD-18co media were enriched with 10 % fetal bovine serum. All incubations in this study were done at a high humidity environment of 5 % carbon dioxide (CO_2_) and at a temperature of 37 °C. The cultured cells were observed and checked daily by using an inversion microscope to see the morphology and cell growth, cultured up to 70-90 % confluence of cells. To subculture the cells, the old media was removed from the flask and phosphate buffer saline (PBS) was used to rinse the excess media. Solution of trypsin-EDTA (0.25 % trypsin/0.03 % EDTA) was added to remove the cells from the surface of the tissue culture flask. Media was added to inactivate the trypsin solution and centrifuged at 1000 rpm for 3 min. The cells were collected and transferred to a new labelled flask with a fresh media. Subculture of cells was done every 2 to 3 days for HCT 116 cell line and 4 to 5 days for CCD-18co cells.

### Evaluation of cytotoxic activity

MTT assay [16] was performed to evaluate cytotoxicity of aqueous, methanol, and acetone extracts at concentration ranging from 12.5 μg/ml - 200 μg/ml at 24, 48 and 72 h against HCT 116 cells as well as acetone extract at 24, 48 and 72 h against CCD-18co. The cells were counted to achieve a concentration of 5.0 x 10^4^ cells/ml. A total volume of 200 μl of cell suspension was seeded in each well of the 96-microtiter plate. The seeded cells were incubated for 24 h before they were treated with each of the extract. Menadione was used as a positive control whereas the untreated cells comprised the HCT 116 cells in the media was used as negative control. A total volume of 200 μl of sample treatment was added in each well and incubated for 24, 48 and 72 h prior to addition of 20 μl of MTT (3-(4,5-dimethylthiazol-2-yl)-2,5-diphenyltetrazolium bromide) at 5 mg/ml to each of the well and left for 4 h incubation. About 200 μl of DMSO solution was added to each well to dissolve the formazan crystals at room temperature and was then incubated for 15 min. The plate was shaken on an automatic mixer for 5 min and the absorbance was read by using an ELISA plate reader at a wavelength of 570 nm. The percentage of cell viability was calculated using the formula given below:

$$ \mathrm{Cell}\ \mathrm{viability}\ \left(\%\right) = \kern0.5em \frac{\mathrm{Mean}\ \mathrm{of}\ \mathrm{treated}\ \mathrm{sample}}{\mathrm{Mean}\ \mathrm{of}\ \mathrm{negative}\ \mathrm{control}}\kern0.5em  \times 100\% $$

The percentage of cell viability against the concentration of test compounds was plotted. The half maximal inhibition concentration (IC_50_) which is the concentration of sample that inhibits cell growth was obtained from the plotted graph.

### Determination of mode of cell death

Flow cytometry Annexin V-FITC/PI assay [17] was used to determine the mode of cell death induced by the tested compound. A total volume of 3 ml of HCT 116 suspension cells at 5 x 10^4^ cells/ml were seeded in each well of the 6-well microtitre plate and incubated for 24 h. The cells were treated with acetone extract at 25 μg/ml, 50 μg/ml, 100 μg/ml and 200 μg/ml, then the 3 ml of the treated sample was added into the 6-well microtitre plate and incubated for 48 h. Next, the cells were washed with 1 ml of PBS solution and trypsinized with 500 μl of trypsin-EDTA to be collected into the same centrifuge tube for each well and was then centrifuged at 2500 rpm for 5 min. The supernatant was discarded and the process was repeated 3 times. A total of 200 μl of annexin binding buffer (1x) followed by 5 μl of Annexin V-FITC (eBioscience, Austria) was added into the tube and left for 15 min in ice. Next, 10 ul of propidium iodide (eBioscience, Austria) at 20 μg/ml was added and incubated in ice condition for another two minutes and finally, 200 μl of annexin binding buffer (1x) (eBioscience, Austria) was added into each tube. Lastly, the solution in the centrifuge tube was transferred into a Falcon tube to be analysed by flow cytometry (BD FASCCanto II) with Cell Quest software (BD Sciences, America).

### Alkaline comet assay

Alkaline comet assay [18] was used to assess gentotoxic damage of HCT 116 by acetone extract from *C. odontophyllum* stem bark at IC_10_ and IC_25_ values whereas menadione was used as the positive control at IC_25_ value. A total of 3 ml of HCT 116 suspension cell with concentration at 5 x 10^4^ cells/ml was seeded in each well of 6-well microtitre plate and incubated for 24 h. The cells were then treated with acetone extract and left to incubate for 30 min before all the media in the well was collected into a centrifuge tube. The wells were washed twice with PBS solution and the cells were trypsinized with trypsin-EDTA solution. The solutions were all combined and were centrifuged at 2500 rpm for 5 min. The supernatant in each centrifuge tubes were discarded and this process was repeated. The low melting point agar (LMPA) and normal melting point agar (NMPA) (Sigma Aldrich, USA) were preheated until the agar melted. When the NMPA reached a temperature of 37 °C, a total of 100 μL of NMPA was pipetted onto a frosted slide and a coverslip (50 mm X 22 mm) was placed on top of the slide and was then taken out slowly after the agar hardened. Next, about 80 μL of LMPA was added into each sample tube on top of the prepared NMPA slide and a coverslip was placed slowly onto the two layers of gel. The coverslip was again taken out slowly and was placed in a coplin jar filled with the lysing solution and cooled at 4 °C for 24 h before the slides were taken out and placed on the electrophoresis tank. Electrophoresis buffer was added into the tank to immerse the slide for 20 min to allow the DNA strands to unwind. The electrophoresis process was carried out for 20 min at 25 V and 300 mA after which the slides were rinsed with neutralizing buffer three times for every 5 min. The slides were then stained with 50 μl of ethidium bromide (Sigma Aldrich, USA) at 20 μg/ml. For each slide, 50 single and isolated cells were analysed for any DNA damage under a fluorescent microscope (Biochrom ASYS, UK).

### Statistical analysis

All data were analysed by using SPSS Software Version 22. All data were done in triplicate and were expressed as the mean ± S.E.M. from three different experiments. One way ANOVA was used to measure statistical differences between the mean in all experiments. The statistical difference was indicated with value *p* <0.05.

## Results

### Assessment of cytotoxic activity

**Fig. 1:**
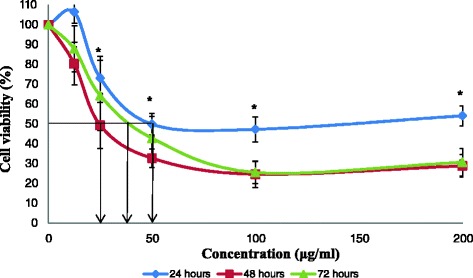
Cell viability of HCT 116 cells after exposure of acetone extract from stem bark of *C. odontophyllum.* Cell survival is expressed as a percentage relative to the negative control (untreated). Acetone extract from stem bark of *C. odontophyllum* at concentration ranging from 0 – 200 μg/ml exhibited cytotoxic activity against HCT 116 cells at 24, 48 and 72 h of treatment exposure. Each point represents the mean of triplicates from 3 different experiments ± S.E.M

**Fig. 2:**
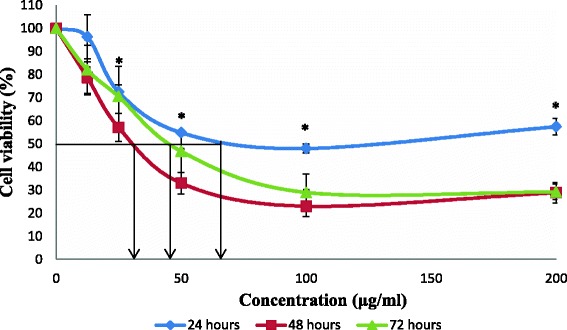
Cell viability of HCT 116 cells after exposure of methanol extract from stem bark of *C. odontophyllum.* Cell survival is expressed as a percentage relative to the negative control (untreated). Methanol extract from stem bark of *C. odontophyllum* at concentration ranging from 0 – 200 μg/ml exhibited cytotoxic activity against HCT 116 cells at 24, 48 and 72 h of treatment exposure. Each point represents the mean of triplicates from 3 different experiments ± S.E.M

**Fig. 3:**
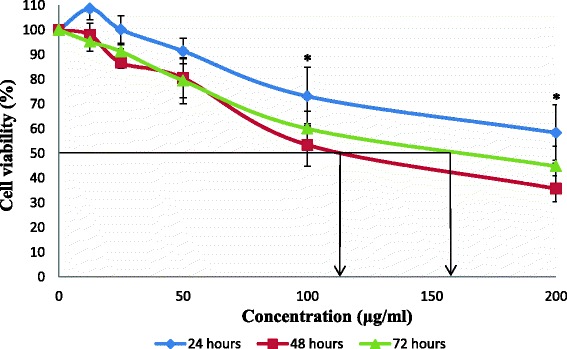
Cell viability of HCT 116 cells after exposure of aqueous extract from stem bark of *C. odontophyllum.* Cell survival is expressed as a percentage relative to the negative control (untreated). Aqueous extract from stem bark of *C. odontophyllum* at concentration ranging from 0 – 200 μg/ml exhibited cytotoxic activity against HCT 116 cells at 24, 48 and 72 h of treatment exposure. Each point represents the mean of triplicates from 3 different experiments ± S.E.M

**Fig. 4:**
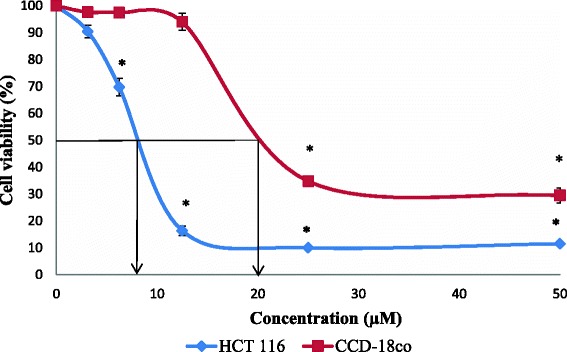
Cell viability of HCT 116 and CCD-18co cells after exposure of Menadione*.* Cell survival is expressed as a percentage relative to the negative control (untreated). Menadione at concentration ranging from 0 – 50 Μm exhibited cytotoxic activity against HCT 116 and CCD-18co cells at 24 h of treatment exposure. Each point represents the mean of triplicates from 3 different experiments ± S.E.M

**Fig. 5:**
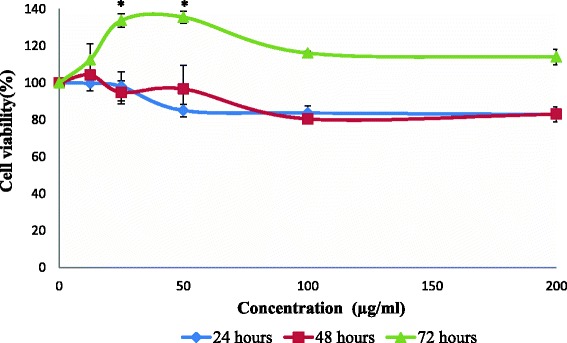
Cell viability of CCD-18co cells after exposure of acetone extract from stem bark of *C. odontophyllum.* Cell survival is expressed as a percentage relative to the negative control (untreated). Acetone extract from stem bark of *C. odontophyllum* at concentration ranging from 0 – 200 μg/ml exhibited no cytotoxic activity against CCD-18co cells at 24, 48 and 72 h of treatment exposure. Each point represents the mean of triplicates from 3 different experiments ± S.E.M

The result for cytotoxic activity of acetone, methanol and aqueous extract from stem bark of *C. odontophyllum* against HCT 116 cells were displayed respectively, in Figs. 1, 2 and 3 and compiled in Table 1. Acetone extract showed a higher cytotoxic effect at IC_50_ value of 50 ± 10.69 μg/ml (Fig. 1) compared with methanol extract at IC_50_ value of 65 ± 2.89 μg/ml (Fig. 2) at 24 h of treatment. However, no cytotoxic effect was observed by the aqueous extract (Fig. 3) at the same treatment duration. At 48 h, the acetone extract displayed the lowest IC_50_ of 25 ± 5.20 μg/ml (Fig. 1) in contrast with methanol extract (IC_50_ 30 ± 4.73 μg/ml) and aqueous extract (IC_50_ 112 ± 6.11 μg/ml) as shown in Figs. 2 and 3, respectively. Table 1 summarised that the IC_50_ values of all three extracts obtained at 48 h were lower than the IC_50_ values at 24 h. When the treatment was prolonged to 72 h, the same profile was observed with acetone, methanol and aqueous extract with IC_50_ values of 40 ± 2.89 μg/ml (Fig. 1), 45 ± 7.64 μg/ml (Fig. 2) and 160 ± 5.46 μg/ml (Fig. 3), respectively. The IC_50_ values obtained at 72 h were slightly higher than the IC_50_ values at 48 h. However, there was no significant difference indicated between these values. Based on Fig. 4, menadione showed prominent cytotoxic activity against both HCT 116 and CCD-18co cells with IC_50_ of 8 μM and 20 μM, respectively. In contrast with the normal colon cell line CCD-18co, Fig. 5 showed no cytotoxic effect by the acetone extract at 24 and 48 h. However, when treatment was prolonged to 72 h, CCD-18co cells proliferated significantly only at the lower doses.

**Table 1:** Comparison of IC_50_ values of extracts against HCT 116

Extract	IC_50_ value		
Time of treatment exposure	Acetone extract	Methanol extract	Aqueous extract
24 h	50 ± 10.69 μg/ml	65 ± 2.89 μg/ml	>200 μg/ml
48 h	25 ± 5.20 μg/ml	30 ± 4.73 μg/ml	112 ± 6.11 μg/ml
72 h	40 ± 2.89 μg/ml	45 ± 7.64 μg/ml	160 ± 5.46 μg/ml

The IC_50_ values of, acetone, methanol and aqueous extract from stem bark of *C.odontophyllum* against HCT 116 cell line at 24, 48 and 72 h of treatment. Data represents the mean of triplicates from 3 different experiments ± error in S.E.M

### Assessment of mode of cell death

**Fig. 6:**
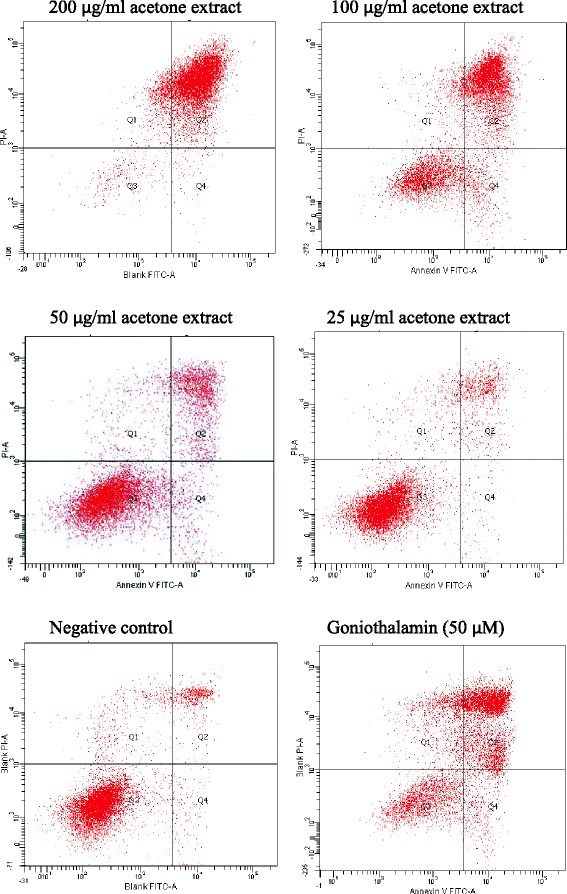
Cytogram of HCT 116 cells induced with acetone extract. Cells were treated with acetone extract from stem bark of *C. odontophyllum* at concentration ranging from 25 – 200 μg/ml after 48 h of treatment

**Fig. 7:**
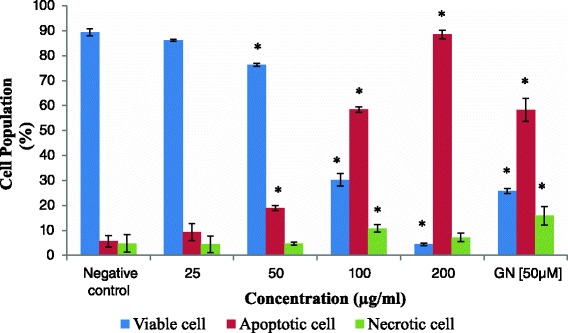
Percentage of cell population of HCT 116 after 48 h treatment. Cells were treated with of acetone extract from stem bark of *C. odontophyllum* at concentration ranging from 25 – 200 μg/ml for 48 h. Each data represents the mean of triplicates from 3 different experiments ± S.E.M

Figure 6 showed the cytogram of HCT 116 induced with acetone extract at 25 μg/ml to 200 μg/ml after 48 h of treatment as determined by flow cytometry using Annexin V-FITC/PI assay. Based on Fig. 7, there is an apparent decrease in percentage of viable HCT 116 cells after 48 h treatment of acetone extract in a dose-dependent manner. One-way ANOVA analysis revealed that there was a significant difference (*p* < 0.05) of percentage of viable cells when treated at high doses (100 μg/ml and 200 μg/ml) of acetone extract which were at 30.3 ± 1.474 % and 4.4 ± 1.68 %, respectively when in comparison with the percentage of viable cells of the negative control (89.367 ± 3.531 %). In accordance with the result, a significant (*p* < 0.05) increased of percentage of apoptotic cells after treatment with acetone extract can be observed at concentration 100 μg/ml (58.9 ± 1.158 %) and 200 μg/ml (88.53 ± 1.763 %) when compared to the percentage of apoptotic cells in negative control (5.667 ± 2.234 %). A significant (*p* < 0.05) increased in necrotic cells can only be observed at concentration 100 μg/ml with a total of 10.8 ± 2.451 % compared to the negative control which wgs at 4.767 ± 1.378 %. The positive control, goniothalamin, displayed significant percentage of viable, apoptotic and necrotic cell population at 25.767 ± 3.733 %, 58.333 ± 4.694 % and 15.9 ± 1.00 %, respectively.

### Assessment of genotoxic activity

**Fig. 8:**
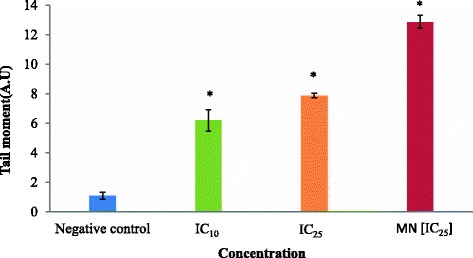
Tail moment of HCT 116 cells after 30 min of treatment. Cells were treated with acetone extract from stem bark of *C. odontophyllum* for 30 min. Each data represents the mean of triplicates from 3 different experiments ± S.E.M

**Fig. 9:**
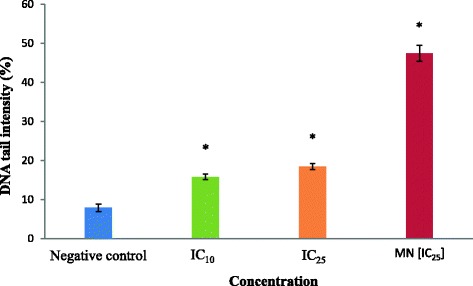
DNA tail intensity of HCT 116 cells after 30 min of treatment. Cells were treated with acetone extract from stem bark of *C. odontophyllum* for 30 min. Each data represents the mean of triplicates from 3 different experiments ± S.E.M

**Fig. 10:**
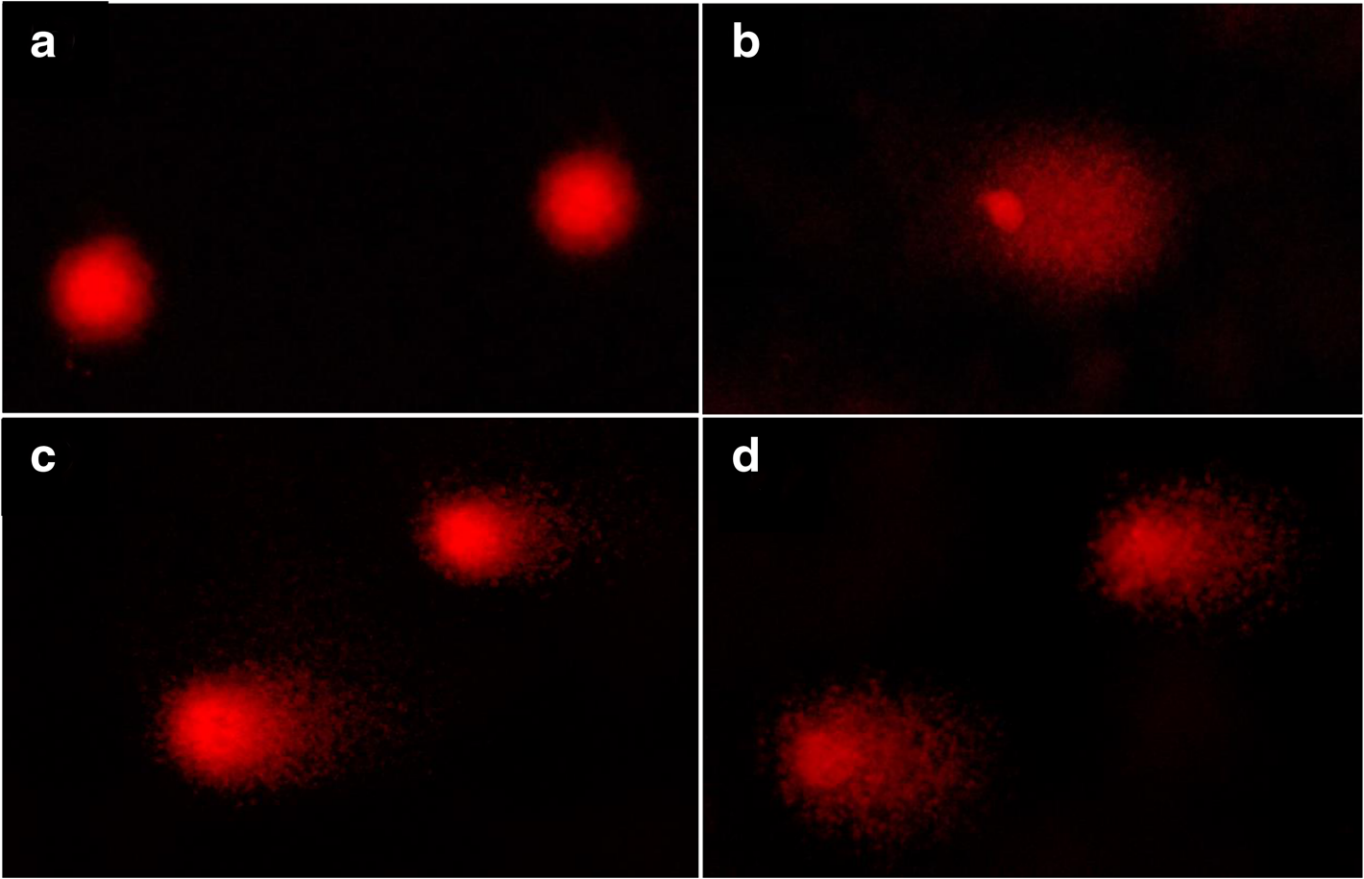
Photo of a single HCT 116 cell stained with ethidium bromide under fluorescent microscope at 30 min. **a** Negative control: untreated cell (**b**) Menadione at IC25 (**c**) DNA migration of HCT 116 cell induced by acetone extract at IC10 (**d**) DNA migration of HCT 116 cell induced by acetone extract at IC_25_

Figure 8 presented tail moment of HCT 116 cells after treatment with acetone extract at 8 μg/ml (IC_10_ value) and 15 μg/ml (IC_25_ value) for 30 min. One-way ANOVA analysis displayed a significant (*p* < 0.05) increased of HCT 116 cell tail moment at both concentration 8 μg/ml (IC_10_ value) and 15 μg/ml (IC_25_ value) when compared to the negative control (1.080 ± 0.239 A.U.) which were 6.187 ± 0.718 A.U and 7.877 ± 0.142 A.U, respectively. Based on the study, the value of tail moment increased as the concentration increased despite no significant difference (*p* > 0.05) between these values. The positive control, menadione, at 5 μM (IC_25_) exhibited significant (*p* < 0.05) tail moment of HCT 116 cells which was at 12.863 ± 0.441 A.U. The percentage of DNA tail of HCT 116 cells after treatment with acetone extract at 8 μg/ml (IC_10_ value) and 15 μg/ml (IC_25_ value) for 30 min can be observed in Fig. 9. According to one-way ANOVA analysis, there was a significant difference (*p* < 0.05) between percentage of DNA tail of treated HCT 116 cells and the negative control (7.866 ± 0.939 %) with values of 15.799 ± 0.673 % and 18.432 ± 0.751 %, respectively. Again, the percentage of tail intensity seemed to increase as the concentration increases, despite no significant difference (*p* > 0.05) between these values. The positive control, menadione, at 5 μM (IC_25_) revealed significant higher (*p* < 0.05) percentage of tail intensity in HCT 116 cells which was at 47.422 ± 2.02 %. In addition, Fig. 10 showed the DNA migration in treated HCT116 cells and no DNA migration was seen in untreated HCT 116 cell (negative control).

## Discussion

The search for a potential anticancer agent has been a challenge for scientists with regards to existence of side effects and drug resistance. Plant-derived anticancer agents are likely to be more effective in the cancer treatment [19]. In this study, evaluation of cytotoxic and genotoxic effects of extracts from stem bark of *C. odontophyllum* against human colorectal cancer cell HCT 116 was done and assessment of cytotoxic effect of acetone extract from stem bark of *C. odontophyllum* against normal colon cell CCD-18co was carried out.

From the MTT assay, acetone extract showed a higher cytotoxic effect compared with methanol extract whereas aqueous extract was not cytotoxic at 24 h of treatment. In the previous study, the same pattern of data was seen by all three extracts but at much higher IC_50_ values [10]. This may be because of the difference in the range of concentration used. The range of the concentration used in this study was smaller compared to the range of concentration used in the previous study. When HCT 116 cells were treated for 48 h, the acetone extract again displayed the lowest IC_50_ value in contrast with methanol and aqueous extract. The IC_50_ values of all three extracts obtained in 48 h were lower than the IC_50_ values in 24 h and this indicated that the extracts gave a time-dependent cytotoxic activity. The longer the exposure of treatment, the lower the IC_50_ value and the higher is their toxic effect [20]. However, when the treatment was prolonged to 72 h, the same profile was observed whereby acetone exerted the lowest IC_50_ value. Although the IC_50_ values obtained in 72 h were slightly higher than the IC_50_ values in 48 h, there was no significant difference (*p* > 0.05) indicated between these values.

From the present study, the methanol and acetone extract at 200 μg/ml produced higher percentage of cell viability than at 100 μg/ml. Exogenous antioxidants that contain in the extract may give both good and bad outcome in redox condition. In addition, a few studies showed that exogenous antioxidants gave debatable results especially at high doses. The type, concentration and matrix of exogenous antioxidant from a natural compound are the features that affect the balance of the benefits [21].

Out of all three extracts, acetone extract exhibited the most potent cytotoxic activity with a significantly higher cytotoxic effect than aqueous extract but the cytotoxicity between acetone and methanol extract showed no significant difference. This finding correlates with the previous study which reported that acetone extract from the stem bark of *C. odontophyllum* [12] displayed the highest cytotoxic effect against HCT 116 cells and was also in agreement with acetone extract from the leaves of *C. odontophyllum* [11]. The cytotoxic activity of a plant against cancer cells is based on their phytochemical properties [22]. The phytochemical screening of aqueous, methanol and acetone extracts from stem bark of *C. odontophyllum* showed that all three extracts contain flavonoid, saponin, tannin, terpenoid and phenolic compounds [12]. The potent cytotoxic effect exerted by acetone extract may be caused by non-polar terpenoid compound. For example, a few terpenoid derivatives from *Rhizoma curcumae* were found to have antiproliferative properties against cancer cell lines [23]. Polyphenolic compounds such as flavonoid may also contribute to the antitumor activity. Flavonoids are known to have beneficial biological effects that include anti-inflammatory, anti-allergic, antimicrobial, anticarcinogenic and antitumor effects [24]. Based from the results obtained, acetone extract was chosen to proceed with the rest of the experiments as it is considered to be the most potent out of all extracts tested.

In general, effective anticancer agents have to execute or halt the cancer cells to live and at the same time will not exert any toxic effect towards the normal cells [25]. As example, extract from *Eugenia jambolana* Lam. are able to inhibit the growth of breast cancer cell line MCF-7aro and MDA-MB-231 but did not inhibit the growth of normal breast cells MCF10A [26]. Generally, antitumor agents show cytotoxicity against cells with higher growth activity by mechanisms such as the inhibition or suppression of increasing nucleic acid synthesis and metabolic pathways than in normal cells [27]. Selective killing towards cancer cells can be achieved by anticancer agents because the characteristics of cancer cells are not the same as the normal cells. Cancer cells are said to be under pressure and are destined to die. They depend highly on abnormalities of apoptosis signalling pathways to stay viable [28]. However, when treatment of acetone extract from stem bark of *C. odontophyllum* was prolonged to 72 h, CCD-18co cells proliferated at the lower doses. This may be due to the presence of compounds that contribute to the mitogenic activity of the cells [29].

Although the IC_50_ value obtained for CCD-18co cells was higher than the IC_50_ obtained for HCT 116 cells, the toxicity of menadione are general to both cells with its Selective Index (SI) value of 2.5. The SI can be achieved by calculating the ratio of IC_50_ value of normal cell and IC_50_ value of cancer cell. SI values that are higher than three are considered as selective toxicity which means that the compound gives selective toxicity to cancer cells but gives no harm or minimal toxicity to normal cells [30]. Hence, we can say that acetone extract from stem bark of *C. odontophyllum* exhibited selective toxicity towards colorectal cancer cell line HCT 116.

Mode of cell death assessment of acetone extract from stem bark of *C. odontophyllum* using flow cytometry Annexin V-FITC/PI labelling assay revealed that the primary cell death of HCT 116 cells was via apoptosis after 48 h treatment. Our findings demonstrated an increase of apoptotic cells and a decrease of viable cells with increasing concentration of acetone extract from stem bark of *C. odontophyllum*. It is said that plant-derived polyphenolic compounds act as antitumor compounds and have apoptosis-inducing properties in cancer cells [31]. Based on the previous study [10], acetone extract from stem bark of *C. odontophyllum* was found to have phenolic compounds and other phytochemicals such as saponin, terpenoid and tannin. A study found that acetone extract from stem bark of *Cephaltaxus griffithii* Hook f. induced apoptosis towards HeLa cells [32]. In addition, the mode of cell death of HL-60 cells after treatment with ethyl acetate extract from stem bark of *Cudrania tricuspidata* was via apoptosis [33]. Apoptosis was also the primary cell death of HCT 116 cells after treatment with ethanol extract from sporophyll of *Undaria pinnatifilda* [34].

The development of plant derived anticancer drug plays a vital role in the treatment of cancer [35]. Many synthetic drugs such as alkylating and antimetabolite agent can affect normal cell and produce side effects to cancer patient [36]. Apoptosis or programmed cell death has been the aim for treatment of cancer at many level of tumour development [37]. During apoptosis, apoptotic bodies undergo phagocytosis and will not submit itself to the inflammation process and may not cause disturbance towards nearby cell [38]. Apoptosis signalling pathways are divided into two mechanisms which are those that involved the mitochondria known as the intrinsic pathway or those that signals through death receptors namely the extrinsic pathway [29]. The specific pathway of apoptosis induced by acetone extract from stem bark *C. odontophyllum* is not fully investigated and understood yet.

Based from the outcomes of MTT assay and flow cytometry Annexin V-FITC/PI assay, the percentage of viable cells obtained were different in these two approaches. This may be due to the difference in the endpoint measurement of these assays [39]. MTT assay is based on the involvement of active mitochondria in living cell to produce succinate dehydrogenase enzyme in order to reduce MTT salt to formazan whereas flow cytometry Annexin V-FITC/PI assay is based on the detection of exposed phosphatidylserine on the outer part of the membrane in dead cells [16, 17]. Besides that, MTT assay can only measure living cell but not dead cell. Flow cytometry Annexin V-FITC/PI assay is able to measure percentage of both living and dead cell in a known amount of cell. The differences between these two assays may be contributing to the deviation of result obtained.

Cytotoxic effect induced by acetone extract from stem bark of *C. odontophyllum* towards HCT 116 cells may be caused by DNA damage and the detected genotoxicity might be the early mechanism of cell death via apoptosis. Alkaline comet assay was used to detect genotoxic effect of tested compound by measuring its DNA damage at single cell that can be observed under a fluorescent microscope whereas a comet head (nucleus) and its tail (DNA fragments) can be seen [40]. The concentrations used in this study were at IC_10_ and IC_25_ values that were obtained from the graph of cell viability versus concentration for acetone extract. A lower and non-cytotoxic concentration was used to dodge any false positive result of dying or dead cells [41]. Among the frequently used comet parameters, percentage of DNA in tail and tail moment could offer the most precise result for the degree of damage [42].

In this study, IC_10_ and IC_25_ of acetone extract from stem bark of *C. odontophyllum* showed significant (*p* < 0.05) DNA damage in HCT 116 cells after 30 min of treatment. DNA is the key target by most cytotoxic anticancer drugs whether it acts directly through reactive metabolites or indirectly through the incorporation into DNA nucleotide analogues or by blockade of DNA-metabolizing functions such as DNA polymerase or topoisomerase. Cancer cells divide more repeatedly than normal cells and this cell division becomes the aim for anticancer agents where the most vital cell cycle phase is DNA replication. Most anticancer agents cause highly damaged-DNA in cancer cells and these cytotoxic agents have different mechanism of action and different types of DNA lesion [43]. Damage may cause disruption of transcription or replication of the DNA where it can lead to cell death or aging [44]. An example of genotoxic agent that binds to DNA and abrupts the replication is doxorubicin. Doxorubicin binds to DNA through intercalation between specific bases and thus prevents DNA synthesis [45]. Nonetheless, the mechanism and type of DNA lesion of HCT 116 cells induced by acetone extract from stem bark of *C. odontophyllum* need further investigation to truly understand the mechanism of DNA damage that leads to apoptosis.

## Conclusion

In conclusion, acetone extract from stem bark of *C. odontophyllum* exerts the most potent cytotoxic effect compared to aqueous and methanol extracts towards human colorectal cancer cell line HCT 116 with no cytotoxic effect against human colon fibroblast cell line CCD-18co. Acetone extract demonstrated cell death through apoptosis and DNA damage was observed in colorectal cancer cell line HCT 116. As a consequence, it has potential in the development of anticancer agent against colorectal cancer.

## Abbreviations

DNA: deoxyribonucleic acid
FITC: fluorescein isothiocyanate
IC_50_: half maximal inhibitory concentration
LMPA: Low melting point agar
MTT: 3-(4,5-dimethylthiazol-2-yl)-2,5-diphenyltetrazolium bromide
NMPA: normal melting point agar
PI: propidium iodide
